# Slotted Photonic Crystal Sensors

**DOI:** 10.3390/s130303675

**Published:** 2013-03-15

**Authors:** Mark G. Scullion, Thomas F. Krauss, Andrea Di Falco

**Affiliations:** SUPA, School of Physics and Astronomy, University of St Andrews, St Andrews, Scotland KY16 9SS, UK; E-Mails: tfk@st-andrews.ac.uk (T.F.K.); adf10@st-andrews.ac.uk (A.D.F.)

**Keywords:** slotted photonic crystal, biosensor, photonic crystal, slot waveguide, microfluidics, label free

## Abstract

Optical biosensors are increasingly being considered for lab-on-a-chip applications due to their benefits such as small size, biocompatibility, passive behaviour and lack of the need for fluorescent labels. The light guiding mechanisms used by many of them results in poor overlap of the optical field with the target molecules, reducing the maximum sensitivity achievable. This review article presents a new platform for optical biosensors, namely slotted photonic crystals, which provide higher sensitivities due to their ability to confine, spatially and temporally, the optical mode peak within the analyte itself. Loss measurements showed values comparable to standard photonic crystals, confirming their ability to be used in real devices. A novel resonant coupler was designed, simulated, and experimentally tested, and was found to perform better than other solutions within the literature. Combining with cavities, microfluidics and biological functionalization allowed proof-of-principle demonstrations of protein binding to be carried out. Higher sensitivities were observed in smaller structures than possible with most competing devices reported in the literature. This body of work presents slotted photonic crystals as a realistic platform for complete on-chip biosensing; addressing key design, performance and application issues, whilst also opening up exciting new ideas for future study.

## 1. Introduction

Small optical biosensors form a substantial part of the growing ‘lab-on-a-chip’ (LOC) paradigm. LOC epitomises the main goal of much biosensor research, that being the ability to shrink down many of the analytical capabilities of a biomedical research lab into a small disposable chip. In one scenario, we can imagine a small chip into which a single drop of blood is placed. This drop is then rapidly screened by a multitude of different sensor elements on the device for many different diseases or other important factors. The full breakdown of the sample is then read-out to the user (for example, a doctor), who can react accordingly. If the device could be made cheap, simple to use and disposable, then it could be kept sterile and would be suitable for rapid, high throughput testing rather than labour intensive laboratory tests, which require much experience, time and training. In essence, the goal is to make devices for detecting biomedical material that are similar to the home pregnancy tests available today: cheap and simple to use, and providing a clear result that can be obtained and read by a non-specialist.

Several challenges are evident to make this vision a reality, but many technologies offer potential solutions to the problem. A growing presence in this area is that of integrated optics, which combines several micro- or nano-photonic components on the same chip, using fabrication techniques borrowed from the electronics industry. The small size of each component means that hundreds of devices be fabricated on the same chip, offering multi-functionality and multiplexing capabilities. Many micro-photonic devices have been proposed as optical biosensors, such as ring resonators [1–3], surface plasmons [4–11], interferometers [12–15], slot waveguides [16–19] and photonic crystals [20–34]. Whilst micro-photonic devices, by definition, are very small, many of them require bulky external support equipment, such as sources and spectrometers, to function fully as sensors. A key challenge is to integrate all of these functions on-chip (lab-on-a-chip), rather than the current reality of chip-in-a-lab.

Some desktop-sized devices containing photonic chips and microfluidic circuits have been developed commercially, such as the surface plasmon based Biacore [35] and the ring resonator based Genalyte [36]. Whilst these devices are very sensitive, they are housed with bulky and expensive instrumentation and are thus not truly lab-on-a-chip. Performing all functions on a chip engenders stability, good alignment and compactness, thus the environmental requirements may not be as challenging as for the above machines. Another issue is getting the target molecules to the sensing region of the device. Andreas Manz and co-workers pioneered the field of Micro Total Analysis Systems (MicroTAS) [37], combining electrical and optical components with small capillaries that can be used to control different liquid samples. Groups such as his, Stephen Quake's [38], George Whitesides’ [39] and others have further developed these microfluidic circuits, allowing a variety of micro-scale chemistry and biology experiments on-chip.

A new player in the field of optical biosensors is the slotted photonic crystal [40–51]. Slotted photonic crystals are typically fabricated in silicon-on-insulator substrates using lithographic techniques, and combine two types of photonic structures, namely the slot waveguide and the photonic crystal to squeeze light down into very small volumes of air. This strong confinement of light in air is the key advantage of the slotted photonic crystal, as it promotes strong light-matter interactions with a substance of interest. Many of the other photonic sensors mentioned above utilize only the evanescent tail of the optical mode for detecting biomolecules, which limits their sensitivity. As shall be seen, the small footprint of the slotted photonic crystal device, as small as a few microns, not only presents opportunities for dense arrays of sensing elements in lab-on-a-chip applications, but also provides potential for some unique and interesting experiments with biological material. The principles behind these devices, and demonstrations of biosensing, are reviewed below.

The review is structured as follows: in Section 2 the principle of operation of optical biosensors, slot waveguides, photonic crystals and slotted photonic crystals are explored and compared. Section 3 presents the general fabrication and characterization protocols. Coupling and propagation losses are investigated in Section 4, whilst Section 5 looks at the integration of slotted photonic crystals with microfluidics. Detection of proteins using slotted photonic crystals is shown in Section 6; before the key challenges facing photonic biosensor technologies are discussed in Section 7 and a summary and outlook at the end.

## 2. Principle of Operation

### 2.1. Optical Biosensors

Optical sensors function by observing changes in some property of light as it passes through the substance of interest. Many optical sensors rely on a spectral feature, usually a sharp peak or dip, which is formed by a resonance (e.g., a cavity). Sensitive refractive index measurements can be made by tracking the peak wavelength of the resonance. In order to make the sensor more specific to a target biomolecule, the surface is coated with antibodies via chemically activated functional groups. These surface coated antibodies act as specific capture agents: protein binding to these surface receptors induces a response from the sensor. When a sample is flown across such a functionalized sensor, in addition to a bulk refractive index change, there will also be a *surface* refractive index change due to the antigen-antibody binding. In this case, the response curve of the device is shaped by the presence of target molecules within the sample. As they diffuse in solution, some of the target molecules will be captured by the functionalized surface receptors, until some saturation level is reached. Higher concentrations of target molecules induce sharper diffusion curves, with higher plateaus. The concentration of binding antigens can therefore be estimated from the shape of this curve. Other antigens cannot bind to a particular antibody as they lack the correct geometry, and thus do not induce the surface refractive index change. This way the sensor response can be attributed to one antigen type alone and is not confused by the presence of multiple antigens in a single solution. Optical biosensors are distinguished from simple optical refractive index sensors by this mechanism, and it is the binding dynamics and the density of surface bound material we measure rather than just a bulk refractive index change.

### 2.2. Slot Waveguides

First proposed in 2004 by Almeida and Barrios at Cornell University, slot waveguides [52] have seen much interest in a number of applications due to their unusual optical properties. They allow strong spatial confinement of light within a narrow air slot inside a material of high refractive index. Standard optical waveguide designs rely on total internal reflection as a means of guiding and confining light. This phenomenon requires for the waveguide cladding to be of lower refractive index than the waveguide itself. The case of the slot waveguide, where light is confined inside a lower refractive index medium, therefore seems a bit strange. This can be explained, however, by the imperfect spatial confinement of light by total internal reflection.

Light incident on a boundary between two non-absorbing dielectrics of permittivity *ε_1_* and *ε_2_* experiences a combination of transmission and reflection effects. The Maxwell boundary conditions require that the tangential components of the electric field **E** and the magnetic field **H**, and the normal components of the electric flux density **D** and the magnetic flux density **B**, be continuous across the boundary in order to satisfy the conservation of energy. As **D =***ε***E**, the boundary condition for the electric flux density can be expressed in the form [53]:

(1) $$[\varepsilon_{1}{\text{E}}_{i0}+\varepsilon_{1}{\text{E}}_{r0}-\varepsilon_{2}{\text{E}}_{t0}]\cdot \hat{\text{n}}=0$$

where **n̂** is a unit vector normal to the boundary and *i*, *r* and *t* subscripts refer to the incident, reflected and transmitted fields respectively, and the 0 subscript refers to the maximum value. In the case of the electric field being parallel to the plane of incidence the normal components can be shown to be [52]:

(2) $$\frac{E_{tn}}{E_{in}-E_{rn}}=\frac{n_{1}^{2}}{n_{2}^{2}}$$

where $n=\sqrt{\varepsilon }$ (for a dielectric). The normal component of the electric field just inside *n_2_* (the low index) is therefore larger than that just inside *n_1_* (the high index) by a factor of $n_{1}^{2}/n_{2}^{2}$.

For total internal reflection, the transmitted angle in Snell's Law becomes complex in nature, with a purely imaginary cosine angle, and the transmitted wave propagates only along the interface [53]. The purely imaginary nature of the normal component (*i.e.*, the cosine angle) of the transmitted field means that it decays exponentially from the maximum value in Equation (2) into the cladding from the boundary. This field is said to be evanescent in nature. High refractive index contrast boundaries can result in quite a large discontinuity in the normal component of the electric field inside the low index medium, for example at an air/silicon interface, the discontinuity immediately inside the air would be almost a factor of 12 greater [52].

**Figure 1.**
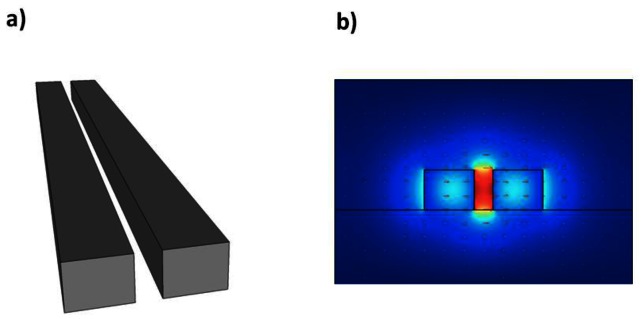
(**a**) Slot waveguide geometry. A narrow air slot is introduced into a ridge waveguide. Alternatively, this can be viewed as two separate waveguides separated by a small region of air. Light is guided mostly in air; (**b**) Field distribution of a slot mode.

If we now consider two optical waveguides in close proximity such that these field discontinuities can interact, which necessitates their separation to be within the decay length of the evanescent field, then these two discontinuities can combine and be enhanced within the narrow air region separating them. Two closely spaced waveguides, or equivalently a narrow slot in a single waveguide, can therefore form a mode of propagation in which most of the light intensity is contained within the low refractive index region, even though total internal reflection is used for guiding. The sharp evanescently decaying discontinuities at each slot wall combine to produce high levels of field inside the air slot as shown in Figure 1(b). This is the basis behind the operation of slot waveguides.

The slot architecture allows for increased light interactions with biomolecules, as the majority of the modal field interacts with the analyte, rather than just the tail as in the standard waveguide geometry. Currently, many slot waveguide biosensors are based on ring resonators. In one such case tested by Barrios and co-workers [16,17] a slot ring resonator is fabricated in silicon nitride and functionalized to detect BSA, the lowest limit being 0.042 μg/mL. A similar device featuring an array of slot ring resonators has been combined with microfluidics, splitters, a photodiode array and grating couplers, and has shown detection of anti-BSA binding (0.125 μg/mL limit) [18]. A smaller biotin coated silicon-on-insulator slot ring [19] that was used to detect avidin at 100 μg/mL concentration has also been reported.

Slot waveguides have also been used to demonstrate optical guiding of sub-100 nm particles along the slot [54]. Whilst this is not strictly ‘trapping’, trapping of sub-100 nm dielectric particles may be possible using slot cavities (e.g., slotted photonic crystals). Trapping of such small dielectric particles is difficult to achieve with more conventional optical traps due to the low polarisability of dielectrics; though slots provide a strong intensity gradient that could compensate for this. In addition, slot waveguides have been used as sensitive refractive index sensors. In one case a refractive index changes as small as 10^−4^ RIU caused by the presence of acetylene gas was demonstrated by Lipson's group at Cornell by incorporating a gas flow cell onto the chip [55].

### 2.3. Photonic Crystals

First proposed by Yablonivitch [56] and John [57], but with origins stretching back as far as Bragg and Rayleigh, photonic crystals have been the centre of much research in integrated optics due to the control of light they offer. In such structures, light can be confined to high quality factor cavities, directed, split or even slowed down. Photonic crystals consist of periodic arrangements of material of different dielectric constant. The physics describing the behaviour of light within them is in many ways analogous to the behaviour of electrons in semiconductors. In both cases, it is the periodicity of the structure that is most important: a periodic structure can only support certain wavelengths. In the case of semiconductors it is a periodic arrangement of atoms, and hence electric potential, that results in the formation of allowed electronic bands. Similarly, for photonic crystals the periodic arrangement of dielectric material results in the formation of allowed photonic bands. In its simplest form, the one-dimensional case, the photonic crystal is recognizable as a Bragg mirror.

Two dimensional photonic crystals [58] are typically created using a triangular or square lattice of air or silica filled holes in a semiconductor medium such as silicon or gallium arsenide. Like the electrons in a semiconductor, photons in a photonic crystal can be thought to occupy certain energy bands corresponding to allowed modes of propagation in the crystal lattice. Two continuums of modes, one corresponding to air (or low index) and the other to dielectric (or high index) guided modes, are separated by a bandgap region. Those frequencies which satisfy the Bragg condition lie within this photonic bandgap region, and are forbidden to propagate through the lattice. Like the 1-D Bragg mirror, 2D photonic crystals can be thought of as very efficient wavelength dependent mirrors, with frequencies within the bandgap being strongly reflected. The strong wavelength dependence of these ‘mirrors’ give the photonic crystal its key functionality. Carving defects into the periodic lattice, for example by removing holes, allows defect states to be created within the photonic bandgap. The lattice surrounding the defect still acts as a mirror for these frequencies, thus frequencies which would normally be rejected can be confined to and guided by defect states within the photonic crystal.

**Figure 2.**
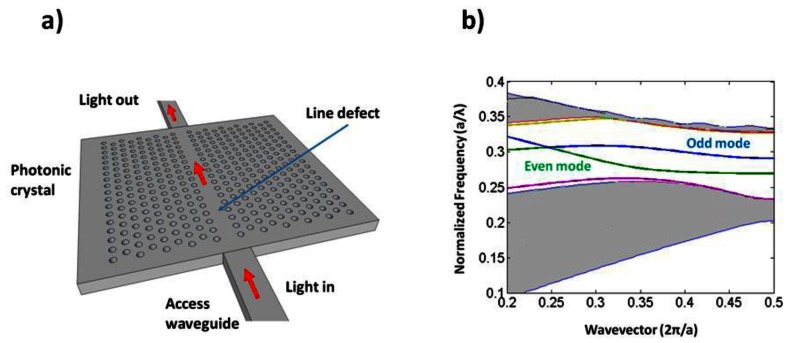
(**a**) Schematic of a 2-D slab photonic crystal, with a W1 defect. Light is delivered to and from a photonic crystal via access waveguides. The defect consists of a single row of holes being removed, which allows normally forbidden frequencies to exist within the crystal. Holes on either side of the line defect behave as very efficient mirrors for forbidden frequencies, and thus can be used to guide light; (**b**) Corresponding photonic band diagram of 2D photonic crystal with W1 defect. Even and odd defect guided modes shown.

One of the most common defects is realized by removing a single row of holes from the crystal, thus creating a **W1** waveguide. This creates the defect modes shown in the photonic bandstructure depicted in Figure 2. Forbidden frequencies can be guided by this defect, while light confinement out-of-plane is provided by total internal reflection.

Whilst slot waveguides specialize in the strong *spatial* confinement of light, photonic crystal waveguides can employ *temporal* light confinement mechanisms such as slow light [59,60]. Physically, slow light behaviour results from coherent backscattering at each unit cell of the photonic crystal forming slow moving interference patterns within the waveguide [59]. The main advantage of slow light is that it results in pulse length compression, which in turn leads to higher intensity and increased light-matter interactions. When more localized defects are utilized (e.g., removal of a few holes), cavities with high quality factor can also be created. In this case a single allowed state is created within the bandgap rather than the extended defect of the W1. Additionally, with careful design, a region of constant, high group index can be realized over a limited bandwidth by engineering the gradient of the photonic crystal's dispersion curve to be relatively flat. The spectral and dispersive properties of photonic crystals can therefore be tailored through structural, rather than material, changes.

The most characteristic spectral feature created by the photonic crystal waveguide is a transmission cut-off near the bandgap. This feature is sensitive to the refractive index of the cladding, and can therefore be used as a point of reference for measuring wavelength shifts resulting from the binding of biomolecules to its surface. For example, detection of single-stranded DNA as low as 19.8 nM has been reported by tracking spectral features near the band edge of a photonic crystal waveguide [20], whilst photonic crystal waveguides have also been used to demonstrate the detection of 10 μg/mL (0.15 μM) BSA by measuring shifts in the cut-off wavelength [21].

By introducing defects into the lattice, high quality factor cavities can be created of a size comparable to a single wavelength of light. High quality factor cavities have sharper spectral features than the photonic crystal waveguide mode cut-off, allowing smaller wavelength shifts to be resolved more easily. Several photonic crystal cavities have been used to demonstrate detection of biomolecules such as BSA (2% [22,23], 1 μM [25]), anti-biotin (20 pM [24]), biotin (3 μg/mL [28], 1 nM [29]) and 3-APTES monolayers [26]. The small size of the photonic crystal cavity is a big advantage over the larger microring cavities, as it gives greater free spectral range (typically ∼100–200 nm for the crystal *versus* ∼1–10 nm for the ring), allowing many more devices of different resonance to be multiplexed. In one example of this, arrays of 1-D photonic crystals with cavities were used to demonstrate detection of anti-streptavidin (1 μg/mL limit) [27]. In this demonstration, each cavity of unique resonance is excited by a single bus waveguide, allowing the response of multiple cavities, each optimized for a different target biomolecule, to be obtained from a single spectrum. 1-D photonic crystals can also be constructed from porous silicon, which is used in many different biosensor platforms [61] due to its large surface area. In one such device [30], protease concentrations as low as 14 μM could be detected by the human eye (7 μM with a sensor) due to colour changes in the photonic crystal as a result of infiltration into the pores.

Photonic crystal structures can also support guided resonances [62], in which light is coupled to a short lived guided mode. Such modes are leaky as they exist above the light line and can therefore couple out of the slab; so guided mode resonance devices can be used at normal incidence. The resonances can result in sharp spectral features which can be used for sensing. Lin *et al*. [31] exploit such a resonance in a sub-wavelength grating to measure the surface attachment of cells. As the local refractive index changes on the grating due to cell attachment, the position of the spectral feature shifts accordingly. This concept has been used to image the attachment of cells to a surface in detail [32,33], which is useful in ascertaining the viability of a cell. Light is scanned over a range of angles through the grating (and an objective) and onto a CCD camera. From these images, the spectral profile at each point on the cell can be determined with a resolution of 0.61 μm^2^. The device has been incorporated into a 96-well plate and is being developed commercially by SRU Biosystems [63].

### 2.4. Slotted Photonic Crystals

**Figure 3.**
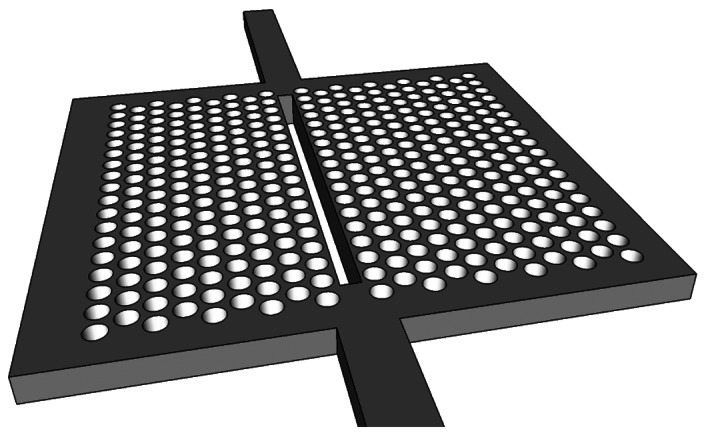
Sketch of slotted photonic crystal geometry. Slot waveguide defect introduced to guiding region of W1 photonic crystal.

**Figure 4.**
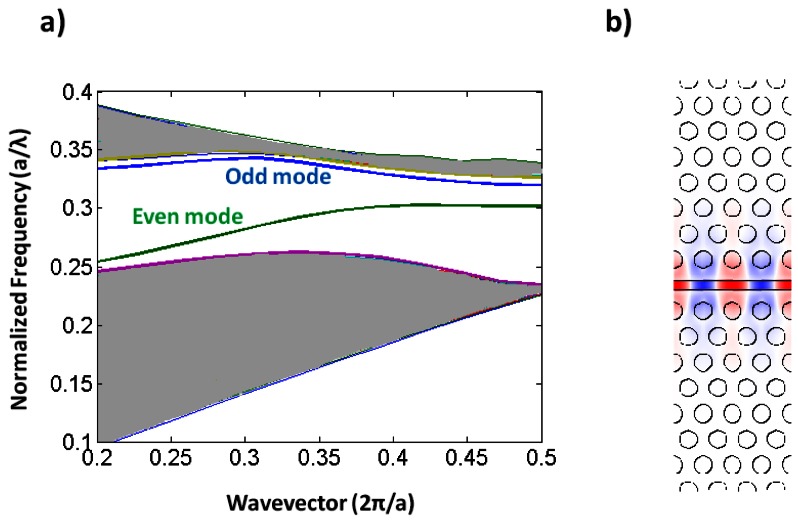
(**a**) Photonic band diagram of 2-D slot photonic crystal; (**b**) Mode profile.

Slotted photonic crystals combine the advantages of spatial confinement of light in air of slot waveguides with the temporal confinement of light provided by photonic crystals in a single structure. This is advantageous because most of the light interacts with the contents of the slot. Simply, a slotted photonic crystal can be viewed as an air slot defect within the guiding region of a standard W1 photonic crystal as shown in Figure 3. Early work in this area [40] has shown, however, that the optical properties of slotted photonic crystal waveguides are very different to that of their non-slotted counterparts. Slotted photonic crystals guide light primarily in air, which is in stark contrast to the conventional non-slotted photonic crystals where most of the light is confined within the high index dielectric. A typical band diagram for a slotted photonic crystal is shown in Figure 4. Comparing Figure 4 with Figure 2, it is evident that the fundamental even modes of the non-slotted and slotted photonic crystals have gradients of opposite sign. This introduces problems associated with efficiently coupling light into the structure, requiring a novel coupler as will be seen later.

Initial work with slotted photonic crystals has shown that they make excellent chemical sensors. Sensitivities of up to 1,500 nm/RIU have been reported, with detection limit 7.8 × 10^−6^ RIU, by our group [41]. We note that that this sensitivity is even higher than the predicted 500 nm/RIU due to the wetting properties of the slot. Solutions containing dissolved material have better wettability than pure water. Other groups have shown detection of gases using these devices, in one case using cavities to detect a variety of gases (510 nm/RIU) [43], or by using slow light effects to enhance absorption of light by methane (100 ppm limit) [44]. The same slow light device has also been used to measure xylene in water (86 μg/L limit) [45]. A group at COBRA (Eindhoven, The Netherlands) have used a cavity created by two deflected photonic crystal nanobeams fabricated in InGaAsP substrates to demonstrate detection of sugar/water solutions with up to 900 nm/RIU sensitivity [46]. The device is covered with a single layer of quantum dots, and is excited by a CW laser. Refractive index shifts are detected by measuring changes in the photoluminescence spectrum of the quantum dots.

A few functionalized slotted photonic crystals have also been demonstrated, for example a glutaraldehyde coated slotted photonic crystal nanolaser in GaInASP/InP has been demonstrated to detect BSA with very high sensitivity (410 nm/RIU, 255 fM limit), by observing changes in the laser spectrum [50]. Whilst BSA has a high affinity for glutaraldehyde, this reaction is non-specific, and in this particular case the affinity was calculated to be four orders of magnitude higher than normal. Such an enhancement, the authors postulate, could be because of optical forces aiding the trapping of the biomolecules within the laser cavity. In another device from the University of Rochester, single latex spheres, 100 nm in diameter, are detected using a slotted microcavity photonic crystal [51]. Our group at St Andrews has developed functionalized slotted photonic crystal cavities, along with integrated microfluidic channels, and has shown detection of the protein avidin at concentrations as low as 15 nM [42]. The main advantages over ring-resonators are the compactness and larger free spectral range which allows potential for 100s of sensors to multiplexed on the same chip with different frequencies.

### 2.5. Comparison of Small Optical Biosensors

Many different figures-of-merit are used to assess the performance of biosensors. These include, the minimum detected (or interpolated) concentration (in terms of parts per billion, μM, particles/L or g/L), the bound mass (in terms of pg), the number of molecules or cells, or the surface density (in terms of pg/mm^2^) which makes it difficult to compare different designs. We suggest quoting the minimum concentration detected, as this can be readily checked and controlled, unlike other quantities which rely on assumptions such as the bound mass. This concentration performance will, however, vary between different antibody-antigen pairs, and is not what the sensor physically measures (it measures surface density of bound material). Expressing the concentration in μM gives an idea of the number of detected molecules per litre, but makes molecules of different mass hard to compare; whilst on the other hand using g/L lets you compare different mass concentrations, but not different number of molecules without additional information. Comparing biosensors is therefore not trivial, and the more figures-of-merit can be supplied the better.

The table below shows a comparison between photonic crystal, slot waveguide and slotted photonic crystal biosensors highlighting their size, sensing area, target substance, detection limit, and to aid comparison, sensor response for a given concentration (where the information is available or can be easily derived).

As can be seen from Table 1, slotted photonic crystals offer the most compact solution of the optical biosensors shown. Wavelength shifts comparable to much larger structures can also be achieved. These wavelength shifts are an order of magnitude greater than those of standard photonic crystals. These observations highlight the strengths of combining slot waveguides and photonic crystals in a single structure. The smaller size offered by the temporal confinement within the photonic crystal allows greater free spectral range and potential for dense multiplexing than standard slot waveguides. The increased wavelength shifts results from the strong spatial confinement in air offered by the slot. Bigger wavelength shifts for a given concentration reduce the performance requirements of any potential on-chip spectrometer; a necessary component for true lab-on-a-chip devices. Also evident is that many photonic crystal and slot waveguide based biosensors have demonstrated proof-of-principle by detecting substances such as streptavidin or BSA. Whilst these substances can be conjugated to a vast library of different antibodies [64], in general, most of these devices have yet to find application in a real world problem of importance. This is to be expected, however, due to the relative infancy of many of these devices when compared to the Biacore surface plasmon devices [35] sold commercially. One of most popular of these devices, the Biacore 3,000 is capable of detecting small biomolecules in concentrations below 1 nM, using samples as small as 5 μL, inside four different flow cells which can be optimised for different antibodies. Although the sensor area is on the order of square millimetres, the surrounding equipment result in a large machine that weighs 50 kg and could only be of real use within a lab. Although the recent Biacore 4,000 machine has the ability to run 4,800 interactions in a period of 24 hours, only 20 different target spots (*i.e.*, 20 different assays) can be addressed, which is an improvement over the four of the Biacore 3,000, but a long way off the desired hundreds or thousands in protein microarrays [65]. The goal of much research in biosensor technologies is to produce a cheaper, more compact and higher throughput device that can compete with the performance of the Biacore without being restricted to high-end labs. Making the devices smaller is therefore of great benefit for multiplexing and higher throughput.

There may also be some room for improving experimental performance through better environmental control. Much of the cost for Biacore comes from the great lengths they go to stabilising the sample environment, minimising noise (such as temperature fluctuations) from unwanted sources. Whilst ‘lab-on-a-chip’ devices will also have to take environmental conditions into consideration, the integrated optics approach is perhaps more stable and requires less engineering as all the elements are on the same chip, thus variables can be cancelled out more easily using a reference.

## 3. Fabrication and Characterization

**Figure 5.**
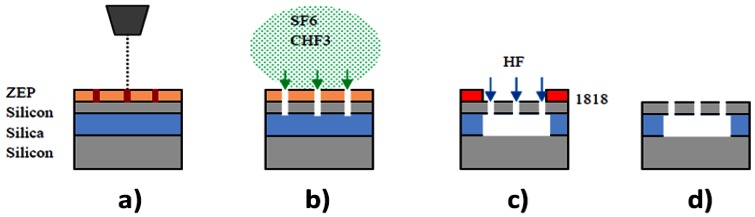
Photonic crystal membrane fabrication steps. (**a**) ZEP e-beam resist coated SOI substrate exposed to electrons in electron beam writer; (**b**) Sample developed exposing surface in pattern of design. Silicon etched using plasma of reactive ions; (**c**) Silica under photonic crystals removed using hydrofluoric acid through an S1818 photoresist patterned window. d) S1818 removed and final membrane sample cleaved.

**Figure 6.**
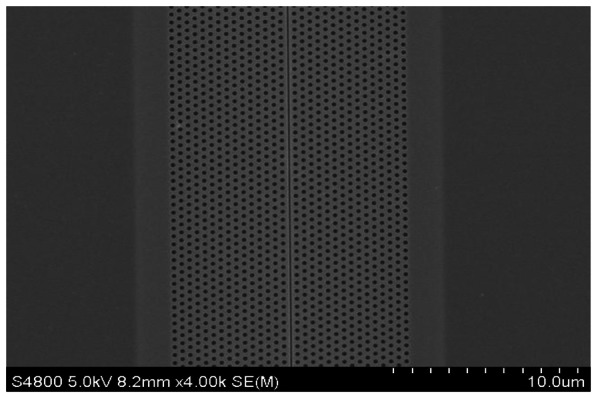
SEM image of slotted photonic crystal fabricated in SOI.

**Figure 7.**
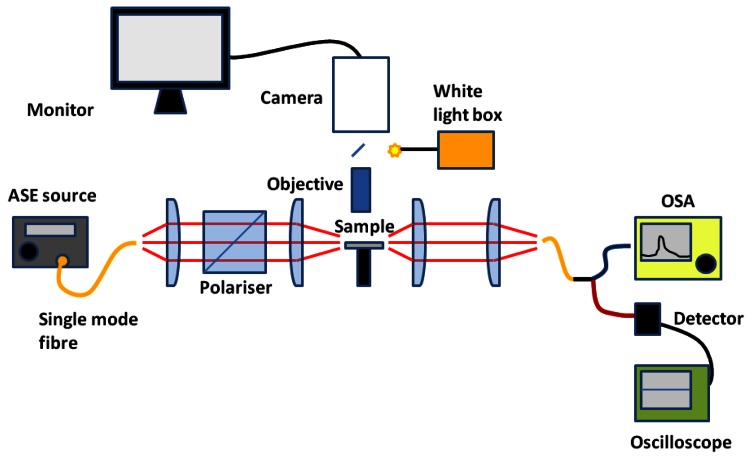
Optical endfire setup for transmission measurements. TE polarised light from broadband ASE source focused onto sample edge facet using aspheric lenses. Light collected from back facet split between OSA and photodetector. Sample using photodetector signal and camera as reference.

Slotted photonic crystals are typically fabricated in Silicon-on-Insulator (SOI), which consists of a 220 nm top layer of silicon, on top of a 2 μm layer of silica on bulk silicon substrate. The silica layer is typically removed by a hydrofluoric acid (HF) wet etch in order to create a suspended membrane, which preserves vertical symmetry and takes advantage of the high refractive index of silicon for the slot waveguide effect. The fabrication procedure is outlined below in Figure 5, more details of which can be found in [66]. In brief, ZEP resist coated squares of SOI are exposed in an ebeam writer, before etching in a custom built reactive ion etching (RIE) device with a 1:1 blend of CHF3 and SF6 gases. The final structure is then cleaved with a diamond tipped scribe. An SEM image of one such structure is shown in Figure 6. The transmission line optical characterization setup is shown in Figure 7.

## 4. Losses in Slotted Photonic Crystals

### 4.1. Coupling Loss

Whilst the unique optical properties of slotted photonic crystals are advantageous in many applications, this uniqueness also creates additional challenges, for example relating to the coupling of light into these structures. As seen earlier, the even modes of standard and slotted photonic crystals have very different dispersive properties; most notably, the gradient of the dispersion curve is of opposite sign. When these differing structures are coupled together, the group velocity mismatch (from the difference in sign) results in coupling into a backwards-propagating mode, resulting in strong reflection at the interface. In addition, there is also a mismatch between the shape of the slotted photonic crystal mode and that of the ridge waveguides used to deliver light to them. To take full advantage of the slotted architecture, a suitable coupler must be found.

For regular slot waveguides, one solution is to reduce the mode shape mismatch by using a sharply tapered ridge waveguide [67]. The same approach has been used for slotted photonic crystals, by firstly coupling the mode from ridge waveguide to slot waveguide via a taper, and then subsequently from slot waveguide to slotted photonic crystal [68]. Although this approach is relatively simple and goes some way to reducing the spatial mismatch of the modes, it introduces multiple interfaces (taper:slot and slot:slotted photonic crystals at both entry and exit) which all need to be optimized. For example, to optimize the slot: slotted photonic crystal interface, one needs to use a tapering of the hole position over several periods. More importantly, however, this design does not take into account the differences in dispersive properties introduced by the presence of the photonic crystal.

Due to the periodicity of a photonic crystal structure, all of its allowed optical modes can be represented using a region in its bandstructure known as the Brillouin zone. Like in solid state physics, the Brillouin zone lies below wavevector values of π/a in the dispersion diagram (see Figure 2 for example bandstructure), this value itself corresponding to the bandgap. Above π/a, the modes are constructed from multiples of the first Brillouin zone wavevectors, thus secondary Brillouin zones and bandgaps result from the periodicity. When waveguides are used to couple light into standard W1 photonic crystals, group velocity matching is ensured through coupling in the second Brillouin zone, rather than the fundamental first, because here the two modes have the same sign of group index [69]. We have found that this is not the case for coupling between slot waveguides and slotted photonic crystals [70].

**Figure 8.**
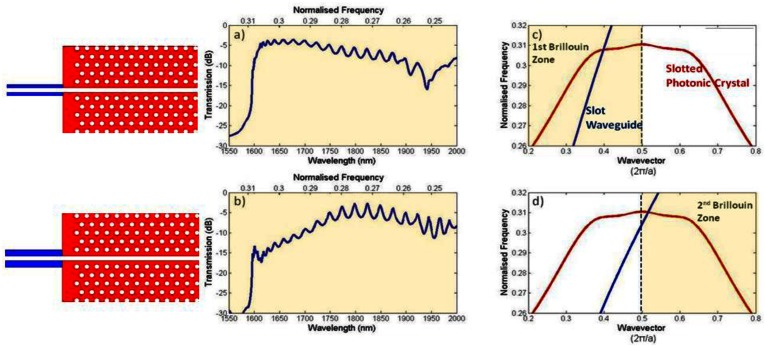
Coupling between slot waveguide and slotted photonic crystal. (**a**,**c**) Coupling in 1st Brillouin zone using narrow slot rails (196 nm wide) results in good transmission due to group velocity and phase matching. (**b**,**d**) Coupling in 2nd Brillouin zone using wide slot rails (294 nm wide) results in poor transmission due to group velocity mismatch. (Copyright © IEEE. All rights reserved. Adapted with the permission from [70]).

Figure 8 shows the guided modes of slot waveguides and slotted photonic crystals, along with simulated transmission spectra for coupling between them, for two different cases. In the first case shown in Figure 8(a,c) for a narrow rail slot waveguide, the guided modes of the slot waveguide (shown in blue) and the slotted photonic crystal (shown in red) intersect within the first Brillouin zone (*i.e.*, below k = π/a). As can be clearly seen, the two modes have the same sign of gradient, and relatively good transmission between the two structures is observed. When the rails of the slot waveguide are made thicker, as in the second case shown in Figure 8(b,d), then the two modes intersect in the second Brillouin zone (*i.e.*, above k = π/a) where they have gradients of opposite sign, and relatively poor transmission between the two structures is observed. In order to ensure favourable coupling to the slotted photonic crystal mode, the rails of the input slot waveguide must be made sufficiently narrow such that intersection occurs within the first Brillouin zone. This introduces limitations on the slots that can be used, and can lead to very fragile practical devices. Another coupling method suggested in the literature is to use multimode interference based structures [71]. In this case a standard input ridge waveguide is locally increased in width at the interface with the slotted photonic crystal. By adjusting the length of this region, the phase difference between the fundamental and second-order modes can be adjusted to give the best mode profile for coupling into the slotted photonic crystal. An enhancement of 20 dB in coupling was achieved for a 35 nm bandwidth [71].

**Figure 9.**
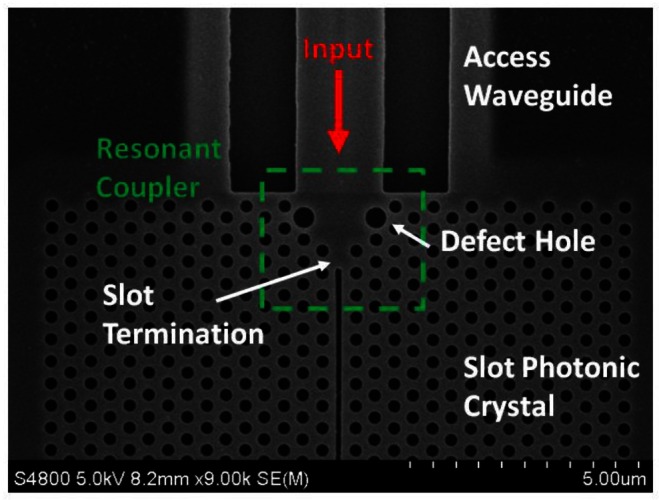
SEM image of slotted photonic crystal with resonant defect couplers and access waveguides, fabricated in SOI.

It has been shown by several authors [72–76] that lattice defects near the edge of a standard photonic crystal can be used to improve their directional emission. One design presented by Bauer and John [77] uses enlarged defect rods at the entrance and exit of a square lattice. Such resonant defects act as secondary sources of light that can cancel out the higher diffraction orders via destructive interference [77]. The result is a self-collimated beam that can be used to achieve very strong coupling with other structures, and operates over a large bandwidth. Recently, in [70] it has been shown that the same scheme can be used for slotted photonic crystals. Using the structure of Figure 9, coupling losses as low as 1.5 dB per interface over a bandwidth of 78 nm were realised experimentally [70]. The performance can be varied depending on the parameters used for the slot termination, the defect holes and the access waveguide width. This solution is robust in terms of fabrication, has good bandwidth and is therefore very suitable for taking full advantage of the slotted photonic crystal design.

### 4.2. Propagation Loss

Recently it has also been shown that the propagation losses of slotted photonic crystals waveguides can be as low as 10 dB/cm [78], and thus approaching similar levels as that of standard photonic crystals [79] and regular slot waveguides [80–83], as shown in Table 2 below. This is somewhat surprising as the surface roughness of the slot introduces another scattering surface that standard photonic crystals do not possess, thus one would expect the losses to be much higher. Broadly speaking, however, the increased scattering on the sidewalls of the slot is balanced by the reduced scattering at the sidewalls of the holes. This balance clearly depends on the slot width, and higher losses occur in narrower slots, as one would expect for the lower proportion of the field on the slot walls.

The propagation losses of slotted photonic crystal waveguides have been shown to be comparable to regular slot waveguides, but the question remains: how low do the losses have to be to be for a useful application? The main advantage of using slotted photonic crystals is that they allow the peak of the optical mode to interact with the contents of the slot. Reducing the losses maximises the amount of light interacting with said contents, which is useful for sensing and other applications. The coupling losses can have a large impact (3 dB *vs.* 15 dB), though this has been rectified using the resonant coupler design. Whilst there is a strong variation in the propagation losses with slot width and wavelength, the structures used are typically less than 100 μm in length. Take for example (a) a slotted photonic crystal with loss of 39 dB/cm, and (b) another with loss of 11 dB/cm. If the structure is only 100 microns long, then (a) results in an absolute loss of 0.39 dB, whereas (b) results in an absolute loss of 0.11 dB. Both of these numbers are quite small when compared to the coupling loss, so do we care? If we use a cavity for sensing, using waveguide (b) allows more passes to be made, increasing the interaction time with the sample and producing a sharper (higher quality factor) resonance. For slow light structures, the losses also become more important due to enhanced scattering resulting from the higher intensities involved. Another point to consider is when several devices are used in series, waveguide (b) allows more to be multiplexed together than (a). It therefore depends on the application. To summarise, for short waveguide structures the propagation losses are not so important, whereas if long optical path lengths are to be used through cavities, slow light or multiplexing, then they can start to become significant. The results also show that the losses are not so much higher than standard photonic crystals to impose restrictions on their use in applications.

## 5. Microfluidic Integration

### 5.1. Background

The previous sections have shown that light can be squeezed down into nanoscale slots, and that this is advantageous for optical sensing; in this section we address the delivery of the sample solution to these sensors. One of the advantages of the slotted photonic crystal sensing architecture is the small footprint, on the order of 10–100 μm, thus potentially hundreds of independent sensors could be fabricated on a single chip. It therefore makes sense to control liquids at a similar scale such that samples can be directed to different sensors, whilst also minimizing their volume; something that can be important with precious samples and reagents. This is where microfluidics [84,85] comes into play; it being the control and manipulation of fluids in flow channels less than 1 mm wide. Microfluidics is a rapidly growing field in its own right, but is also being combined with other disciplines such as biophotonics [86]. One of the key features of fluid behaviour within microfluidic channels is that fluid flow is almost entirely laminar. The small size of microfluidic channels results in viscous forces dominating inertial ones, thus the Reynolds number is always much lower than 2,000, ensuring laminar flow [84].

One of the most common materials used in microfluidic chip fabrication is an elastomeric polymer called poly(dimethylsiloxane), or for short, PDMS. Using soft lithography, microfluidic devices can be rapidly fabricated in PDMS with feature sizes down to 30 nm [87]. PDMS is optically transparent and flexible. This flexibility can be exploited for making components such as pneumatic microvalves and pumps [88] in the microchannels. Here, microfluidic channels are integrated with the slotted photonic crystals as a way to supply the sensors with liquid samples of very low volume, whilst also preventing fast evaporation.

### 5.2. Experiment

To form a master mould with the desired channel design (typically 100–200 μm wide channels), a piece of silicon is cleaned in acetone and IPA whilst inside an ultrasonic bath, and dried in dry nitrogen. SU-8 negative photoresist (2,050:2,000.5 in a ratio of 10:1) is then spun on top of the substrate at 1,000 rpm, giving a thickness of 40 microns. The sample is then baked on a hotplate at 65 °C for 5 min, followed by 95 °C for 15 min. To create the desired pattern, the sample is loaded into a mask aligner and exposed to UV light through a photomask of the channel pattern for 2 min. Following this, the sample is post exposure baked at 65 °C for 1 min and 95 °C for 10 min. The pattern is then developed in EC solvent for 3 min, and finally rinsed in IPA. To ensure good adhesion of the SU-8 to the silicon substrate, the sample is hard baked overnight in a 180 °C oven.

**Figure 10.**
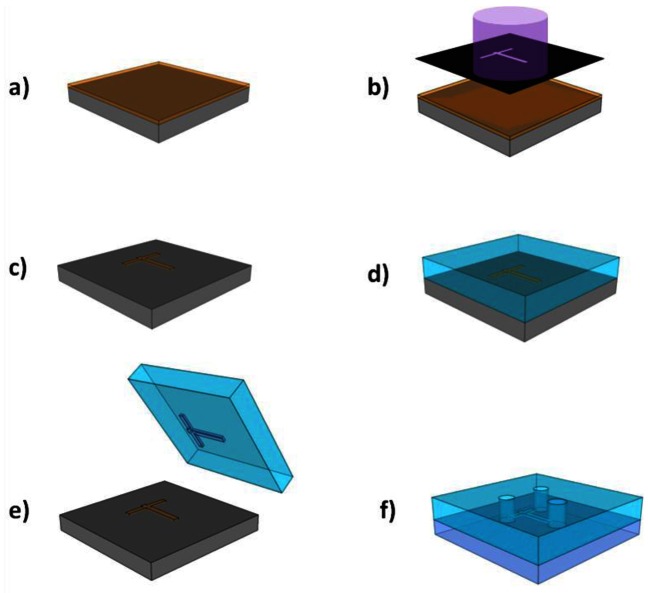
Soft lithography method of fabricating PDMS microfluidics. (**a**) SU-8 photoresist of thickness equal to desired channel height is spun and baked on clean silicon substrate; (**b**) Sample exposed to UV light through photomask of channel design; (**c**) Followed by, post-exposure bake, development and hard baking to complete mould; (**d**) Liquid PDMS base and curing agent poured on top of mould and allowed to solidify; (**e**) Solid PDMS containing imprint carefully peeled from mould. This mould can be used repeatedly for making PDMS imprints; (**f**) Access holes punched in to PDMS in order to connect pipes. PDMS and bottom cover (e.g., glass) then treated with oxygen plasma and bonded together.

The PDMS base and curing agent are mixed in a ratio of 10:1.3 by volume, and degassed in a vacuum chamber to remove bubbles. The mixture is then carefully poured over the master mould inside a plastic Petri dish, and left it at 65 °C for over 4 hours to solidify. The solid PDMS is carefully pealed from the mould and cut to size with a blade. Access holes for tubing are punched through the microchannels where required. To seal the bottom of the channel, a coronal discharge gun is applied to the PDMS stamp with oxygen plasma [89] for 30 s, creating free OH groups on its surface [90]. When placed in contact with another surface with similar free groups, such as oxygen treated glass, PDMS, PMMA or silicon, the two surfaces can form covalent siloxane (Si–O–Si) bonds [90]. Each surface is treated with the oxygen plasma for 30 s, before rinsing in methanol; this allows the two surfaces to be placed in contact and aligned (the methanol acting as a lubricant). To ensure good bonding, this process must be carried out within a few minutes of oxygen treatment, thus methanol is used as it evaporates fast. After contact, the samples are placed in a 65 °C oven for over 4 hours. Better bonds result when using PDMS that is cured on the same day (ideally poured ∼4 hours before bonding). The final sample has tubing of diameter slightly greater than the access holes, compressed into these holes to take liquids in and out of the microchannel. These moulds can be re-used numerous times to make PDMS microchannels. The full process is sketched in Figure 10.

**Figure 11.**
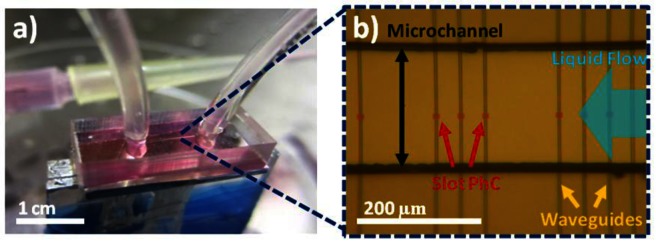
Slotted photonic crystals with integrated microfluidic channels. (**a**) PDMS microchannel bonded on top of SOI chip. Sample glued to glass holder. Tubing inserted into access holes, to which syringes can be connected. (**b**) Optical microscope image of channel with slotted photonic crystals inside. Access waveguides take light to and from photonic crystals. (Copyright © Elsevier. Adapted with permission from [42]).

In order to integrate the PDMS microchannels onto the silicon chip, a slightly different approach is adopted for bonding. Instead of plasma treating both surfaces, a piranha solution is used for the SOI containing the slotted photonic crystals. This method is used due to fears that arcs between a coronal discharge gun and a silicon surface could damage the membraned slotted photonic crystals. After cutting the PDMS chip to size, it is rinsed in IPA within an ultrasonic bath and dried with nitrogen gas in order to ensure the surface is clean. The piece of SOI containing the photonic crystals is then immersed in piranha solution for 15 min, before rinsing in DI water, acetone and IPA to create OH groups on the surface. The PDMS surface containing the microchannel is exposed to oxygen plasma on top of a plastic Petri dish for 30 s using the coronal discharge gun, before rinsing both the SOI and PDMS in methanol. The two surfaces are placed in contact under the microscope, and aligned using a pair of tweezers. The alignment of the microchannel to the photonic crystals has to be completed before the lubricating methanol evaporates, and shortly after oxygen treatment, in order to achieve good bonding at the desired location. Once aligned, the sample is left for a few minutes to allow the methanol to evaporate, before it is carefully transported to a 65 °C oven, and left to bond overnight. After removing from the oven, the sample is bonded to a glass sample holder using silver conductive paint, and tubing inserted into the access holes. Images of a finished chip are shown in Figure 11. No leaks were detected.

## 6. Biosensing Using Slotted Photonic Crystal Cavities

### 6.1. Cavities

Several cavity designs have been explored for slotted photonic crystals, including heterostructure [41,47], local line width modulated [91,92], L3 type [93], and a varying slot width [43] approach amongst others [50,51,94]. Amongst these, the heterostructure approach presents better fabrication tolerances, though all of these can, and have, been adopted for sensing. In the heterostructure cavity of Noda [95] for standard W1 photonic crystals, the lattice is locally expanded in one direction along the W1 (single row of missing holes) waveguide for a few lattice periods. The lattice remains unaltered on either side of this defect. Only light of a certain frequency can be supported in the defect region, and is confined by the surrounding lattice which acts as a mirror. As seen earlier, the different nature of a slotted defect *versus* a standard W1 defect photonic crystal results in different optical properties. To form a cavity in the slotted photonic crystal architecture requires the opposite approach to that of the W1. As shown in [41], heterostructure cavities within slotted photonic crystal require a *compression*, rather than expansion, of the lattice. As the waveguide mode of the slotted photonic crystal cuts-off at high frequency, a new forbidden frequency can be introduced by increasing the frequency slightly into the bandgap, one way being lattice compression. With this design our group has demonstrated theoretical quality factors of 80,000 in air, 6,000 in water, and experimental values of 50,000 in air and 4,000 in water [41].

**Figure 13.**
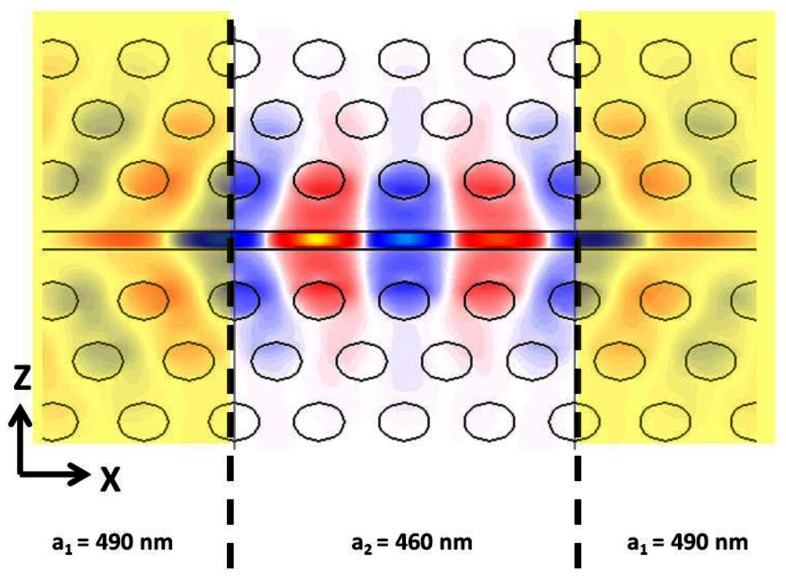
2D FDTD simulation of an air slotted photonic crystal cavity mode showing Ez component. Mirrors are highlighted in yellow. The majority of the field intensity is confined to the slot, a property which lends itself to sensing because of the resulting strong light-matter interaction. (Copyright © Elsevier. Adapted with permission from [42]).

Slotted photonic crystal cavities are advantageous for sensing applications as most of the field interacts with contents of the air slot. This is clear from the previous discussion and is highlighted by the FDTD simulation of Figure 13 for a heterostructure cavity. In conventional photonic crystal cavities and other devices such as ring resonators, most of the light is confined to the dielectric of the waveguide structure, and only the evanescent tail of the optical mode sees the analyte of interest. In the slotted photonic crystal case a change in the refractive index of the slot contents is felt by the majority of the modal field, altering the resonant wavelength of the system more strongly. This field overlap is reflected by the sensitivity 500 nm/RIU *vs.* 50–100 nm/RIU.

**Figure 14.**
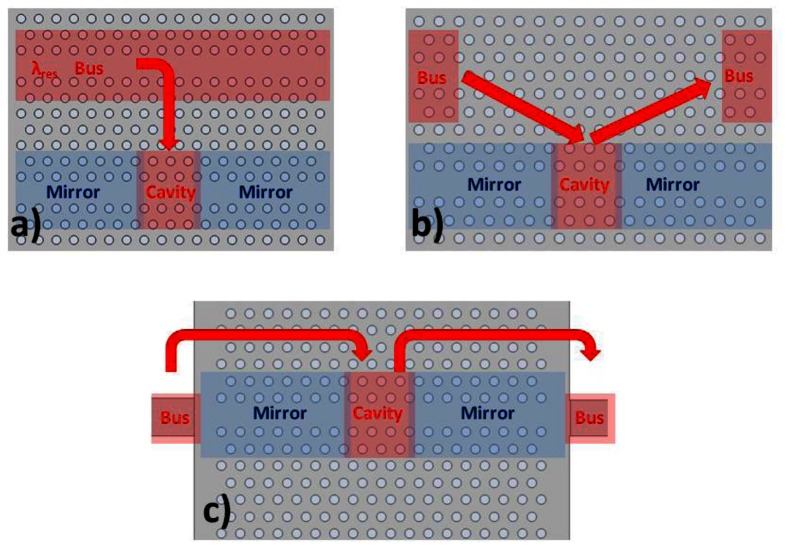
Common photonic crystal cavity coupling mechanisms. (**a**) Side coupling from bus waveguide produces dip in transmission spectrum; (**b**) Side coupling using input and output buses produces peak in transmission; (**c**) In-line coupling produces cavity peak plus guided wavelengths of mirror waveguide region.

We use an in-line coupling mechanism as demonstrated in Figure 14(c). Light of resonant wavelength couples from the mirror interface, into the cavity, and back out to the waveguide beyond the second mirror. This produces a peak in the transmission spectrum. The important difference with side coupling methods shown in Figure 14(a,b) is that other wavelengths of light which are guided, rather than reflected, by the wavelength dependent mirrors can pass also through. This produces a broad region of high signal within the output transmission spectrum, which can be used for alignment purposes. The full spectrum therefore shows a broad region of high transmission, with a stopband at the mirror wavelengths, within which lies the transmission peak of the cavity resonance. The disadvantage of this approach is that there are more interfaces to consider.

**Figure 15.**
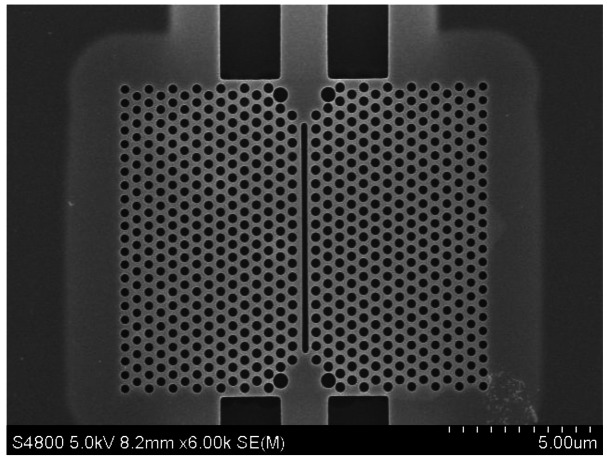
SEM image of slotted photonic crystal cavity fabricated in SOI.

**Figure 16.**
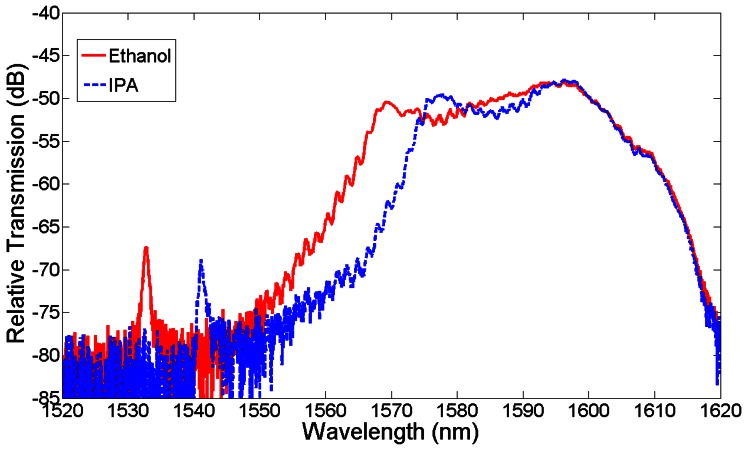
Transmission spectra of a slotted photonic crystal cavity with different alcohols injected into the integrated microchannel.

Cavities were fabricated in SOI as outlined earlier. For operation at 1,550 nm for water filled devices, a hole radius of 150 nm, and slot width of 180–200 nm were chosen. PDMS microfluidic channels of 40 μm height and 200 μm width were integrated with the chip. We found experimentally that most repeatable results occurred when using the interface design shown in Section 4. An SEM image of the structure is shown in Figure 15. As a first test different alcohols were introduced into the microchannel, as shown in Figure 16 below. The cavity peak (around 1,532 nm for ethanol) is clearly distinguished from the bulk transmission of the mirror waveguide (above 1,550 nm for ethanol), and was found to shift significantly with the different alcohols. At 1,550 nm, Ethanol has a refractive index of approximately 1.354 and IPA has 1.378. Given that the shift in resonant wavelength is approximately 10 nm, this corresponds to a refractive index sensitivity of approximately 415 nm/RIU.

### 6.2. Functionalization

In order to test the cavity's potential as a biosensor, suitable capture agents and targets had to be chosen. We initially decided to investigate the avidin-biotin system as it is often used as a model for studying protein interactions [64]. It can also be used to help immobilize a variety of antibodies on surfaces. Its use here is therefore of great interest as it could be adapted to suit most types of antibody, and thus different types of sensor. Biotin can be immobilized on silicon dioxide surfaces using a chemical known as aminopropyltriethoxysilane (APTES). This chemical binds to free OH groups on the surface of the glass, whilst presenting free NH_2_ groups for further functionalization. NHS-linked Biotin can attach to these NH_2_ groups [96]. In order to functionalize the photonic crystals with biotin receptors, a protocol similar to that of De Vos *et al*. [1] was adopted, as they demonstrated biotin functionalization of silicon microphotonic structures.

Before bonding the microfluidics, the SOI chips containing the photonic crystals were immersed in Piranha solution for greater than 20 min to ensure that the surface was clean, hydrophilic, and to expose OH groups on the surface (from the resulting oxidation) to aid functionalization. The microfluidic circuitry was attached as in Section 5. After bonding the chip to a sample holder with silver conductive paint, the chip was placed in the optical characterization setup. APTES was diluted in toluene to a concentration of 1% and was injected into the chip to sit for several hours. We found, however, that the toluene caused the PDMS microfluidics to swell up, and in some cases even melt, destroying both the microfluidic circuitry and the silicon surfaces. The later step of biotin involved DMF, which has similarly detrimental effects on PDMS. As noted by Whitesides’ group [97], PDMS swells up in the presence of nonpolar organic solvents, one good example of which is toluene, with the amount of swelling varying between solvents.

**Figure 17.**
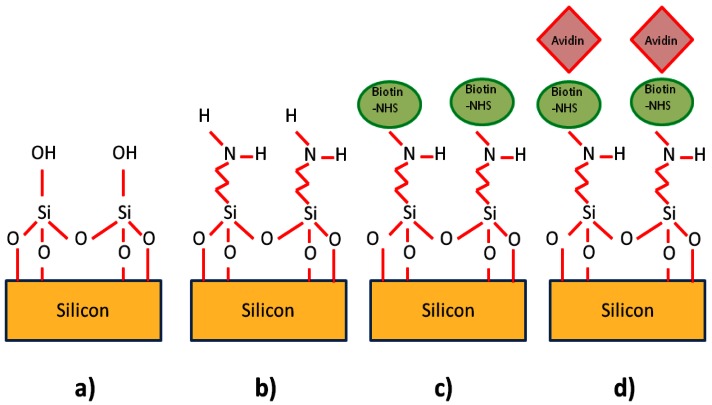
Biotin functionalization of silicon surface using APTES. (**a**) Silicon immersed in Piranha solution to expose OH groups on surface; (**b**) APTES links to OH groups on surface and presents NH_2_ groups; (**c**) NHS linked biotin attaches to NH_2_ groups; (**d**) Avidin captured by immobilized biotin.

In order to combat this problem, we tried a number of approaches. Liquid silica solutions such as Flowable Oxide (FOX) were coated on the PDMS chip through spinning, and baking on a hotplate. The problem with this method is that FOX 14 (Dow Corning) requires baking to temperatures in excess of 400 °C, which are not suitable for PDMS (PDMS is thermally stable only up to approximately 180 °C), thus the full glass transition could not be allowed to take place. Another approach was to sputter thin layers of gold into the channel itself, masking the surrounding PDMS surfaces with tape during the procedure so as to protect the parts to be placed in contact with the silicon. This appeared to reduce the swelling in toluene, but resulted in reduced light transmission, suggesting the gold was scattering some of the light at the edges of the channel in contact with the silicon or that the gold was detaching when solutions were flowed through the channel. As we did not succeed in adapting the PDMS to the functionalization chemicals, we decided to change the chemistry. Literature (for example [2,27,96]) shows that silicon dioxide surfaces can also be functionalized with APTES dissolved in many different solvents including ethanol and water. Some of these solvents only have very little effect on the PDMS. The immersion times also varied widely from a matter of minutes to 24 hours. After several test runs with different concentrations and timing, the protocol described below was adopted (see Figure 17).

Following the piranha and microfluidic bonding steps mentioned above, ethanol was diluted to a concentration of 95% in DI water (1 part DI water to 19 parts ethanol). Using this solution, APTES was diluted to a concentration of 2%. This was then injected into the microchannel and left overnight (typically 19 hours in total). No noticeable swelling or deterioration of the PDMS was detected. After this, the channel was flushed several times with ethanol and then DI water, and the whole chip was placed inside a 65 °C oven for over 5 hours in order to allow the APTES to cure. Biotinamidohexanoic acid N-hydoxysuccinimide (NHS linked biotin) ester powder (Sigma–Aldrich, Dorset, UK) was dissolved in dimethylformamide (DMF) to a concentration of 1 mg/mL. This was then further diluted in phosphate buffered saline (PBS) to a concentration of 100 μg/mL. This was flowed into the cured chip, and left to sit in the channel for over 12 hours in order to saturate the surface with biotin receptors. The chip was then flushed several times with PBS. As each step produced a shift in the cavity peak wavelength that did not recover after flushings, then the functionalization protocol was taken to be successful.

### 6.3. Protein Sensing

**Figure 18.**
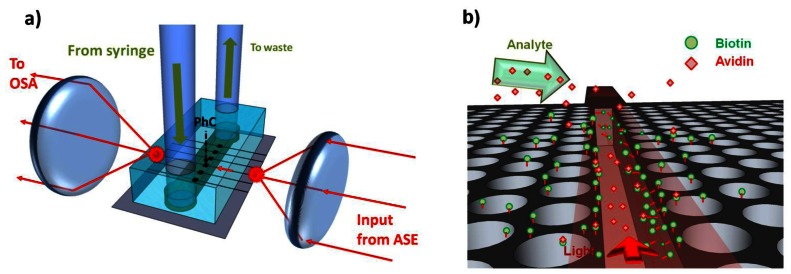
Biosensing experimental setup (not to scale). (**a**) Light focused into optical waveguide at front edge. This light is then taken through the photonic crystal (inside the microfluidic channel) and then to the back edge where it is collected. Liquids are passed through the microchannel via connected tubing and syringe; (**b**) Avidin binds to biotin coated sensor inducing a shift in the cavity peak wavelength.

**Figure 19.**
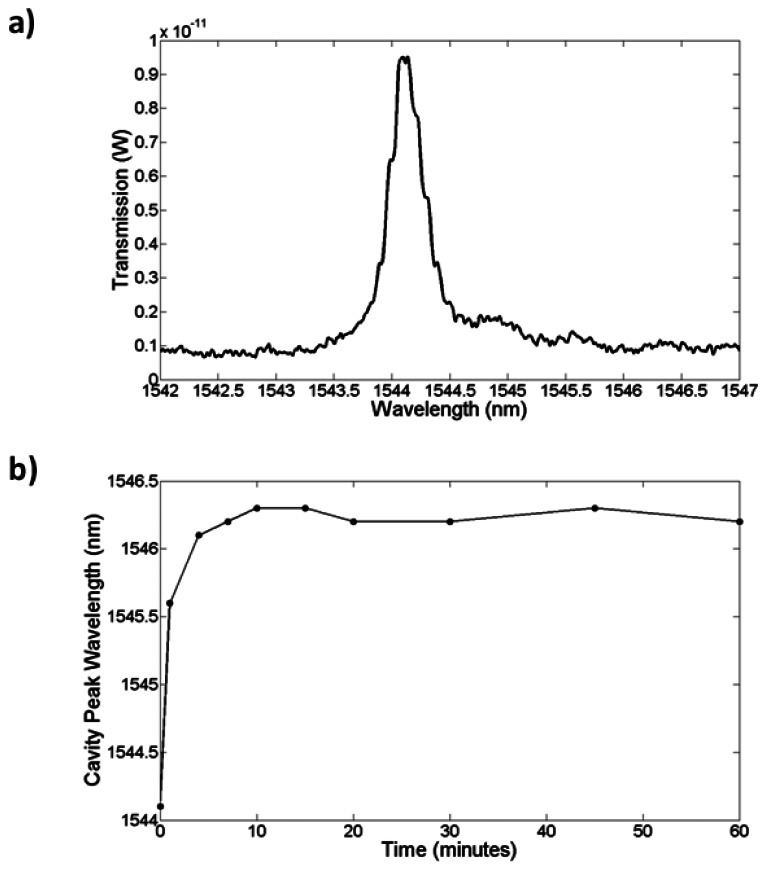
Initial avidin biosensing experiments. (**a**) Cavity spectrum; (**b**) Cavity peak wavelength as a function of time for 10 μg/mL concentration.

To test the performance of the device, avidin from egg-white lyophilized powder (Sigma–Aldrich) was diluted down to 10 μg/mL in PBS. After functionalization and optical alignment, a blank PBS solution was first used, before injecting the avidin, via a syringe and inlet tubing into the chip as shown in Figure 18 (see Section 3 for full optical setup). During this time the transmission spectra of the slotted photonic crystal was recorded from the optical spectrum analyser (OSA). The high sensitivity setting on the OSA was used to minimize noise. The results are shown in Figure 19, which depicts the variation in the peak wavelength of the cavity *versus* time.

**Figure 20.**
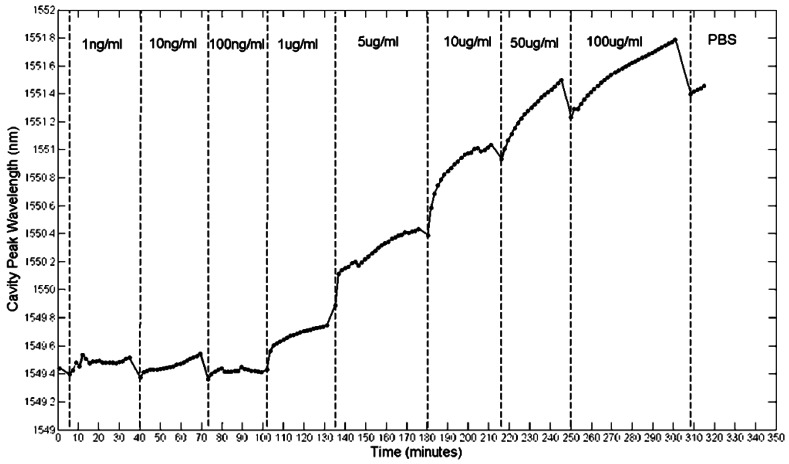
Cavity peak wavelength for a single slotted photonic crystal cavity as a function of time for different concentrations of avidin in PBS (Copyright © Elsevier. Adapted with permission from [42]).

The results of Figure 19 showed that avidin binding produced a large response, and that this response is shaped by diffusion. 30 minutes was also found to be sufficient to reach saturation. In order to test the lowest limit of detection another fresh chip was fabricated and functionalized as before. This time, avidin was serially diluted in PBS from 100 μg/mL down to 1 ng/mL. Each solution was introduced in order of increasing concentration into the chip and left to sit for typically 30 min each. Spectra were taken every minute, and the cavity peak wavelength extracted from a Lorentzian fit. Whilst some errors will result from the fit, given that the shifts in wavelength during binding were greater than the linewidth of the cavity, these fitting errors were assumed to be negligible. The results are shown in Figure 20. Note that all of these measurements were performed on a single photonic crystal.

**Figure 21.**
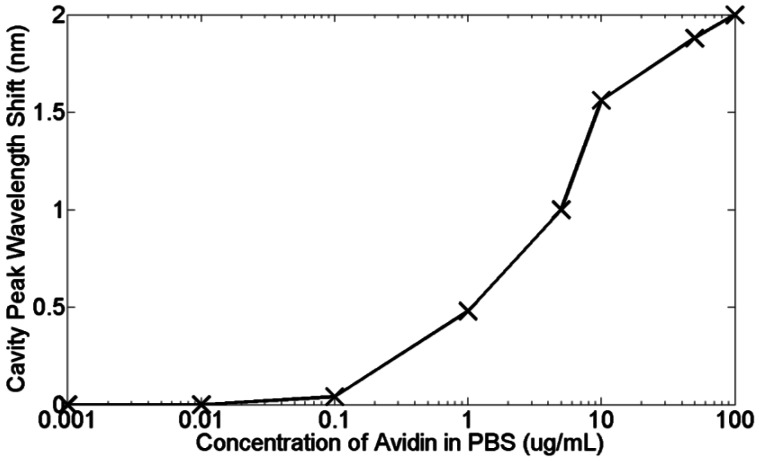
Cavity peak wavelength shift after binding for each avidin concentration. (Copyright © Elsevier. Adapted with permission from [42]).

Figure 20 highlights again that the time-response of the sensor is dominated by diffusion. Whilst the experiment was only run for 30 min per concentration, leaving it for longer would allow these diffusion curves to plateau at their respective saturation values. Each concentration has a different curve and as expected, lower concentrations have shallower gradients. The little dips in sensor response at each injection point can be attributed to the flushing of material that has not properly bonded to the biotin [18]. A better measure of the actual bound material are therefore these dip points, as here the unbound material has been discarded. Taking these points, Figure 21 shows how the sensor response for a thirty minutes reaction varies for different avidin concentrations. The lowest concentration that can be clearly observed is 1 μg/mL. The average quality factor was estimated to be 3,000 from the measurements.

### 6.4. Analysis

**Figure 22.**
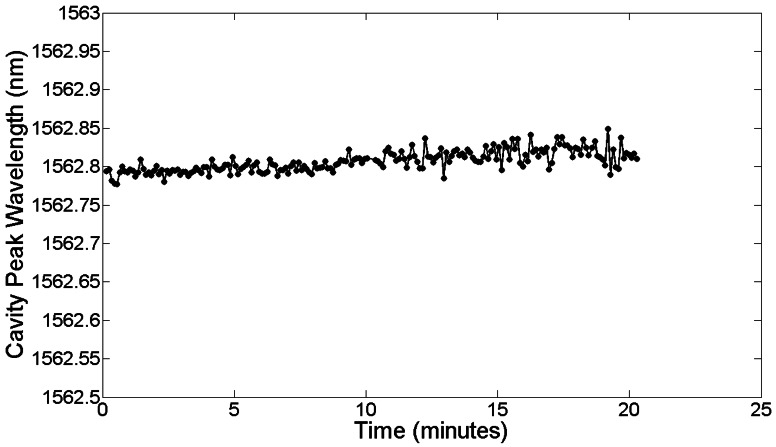
Cavity peak wavelength as a function of time for PBS filled microchannel.

From Figures 20 and 21, a dissolved avidin concentration of 1 μg/mL was clearly detected. As avidin has a molecular weight of 68 kDa [98], this corresponds to a molar concentration of 15 nM. Given that the sample stage was not temperature controlled, thermal noise must be taken into consideration. For de-ionised water at a temperature of 20 °C, light of wavelength 1,550 nm experiences a thermo-optic coefficient of approximately 10^−4^ RIU/°C [99]. Assuming a maximum temperature fluctuation of 0.5 °C within the lab over the time period for each concentration, and the refractive index sensitivity of the slotted photonic crystal cavity being 500 nm/RIU, this would correspond to a shift in resonant wavelength of 0.025 nm. Even assuming an order of magnitude higher thermo-optic coefficient of 10^−3^ RIU/°C would produce a shift of 0.25 nm. In both cases, the wavelength shift is smaller than the shifts observed for avidin concentrations above and including 1 μg/mL. To experimentally test the thermal stability of the PBS solutions, the cavity peak wavelength for a PBS filled channel was recorded over a 20 min period. The results shown in Figure 22 show fluctuations in wavelength of less than ∼0.05 nm, which is consistent with a thermo-optic coefficient of 5 × 10^−4^ RIU/°C. With these numbers, and given the linewidth of the cavity, it is fair to say that binding can be attributed to wavelength shifts of greater than 0.1 nm. Better environmental control should allow this number to be reduced in future. Comparing with Table 1 shows that a detection limit of 15 nM is impressive given the small size of the slotted photonic crystal cavity. To aid comparison further, an estimate of the surface density and bound mass is also made. For the smallest observed wavelength shift *δλ* (in this case taking 0.4 nm for 1 μg/mL) the surface density of bound material *σ_p_* can be estimated from the equation [100]:

(3) $$\frac{\partial \lambda }{\lambda }=\sigma_{p}\alpha_{ex}\frac{2\pi \sqrt{n_{m}^{2}-n_{s}^{2}}}{\varepsilon_{0}\lambda^{2}}\frac{n_{m}}{n_{s}^{2}}S$$

where *α_ex_* is the excess polarizability of the molecule, *n_m_* is the refractive index of the sensor substrate, *n_s_* is the refractive index of the analyte and *S* is the bulk refractive index sensitivity. Taking the values for this experiment: *α_ex_* = 4πε_0_ (3.2 × 10^−21^) cm^3^[101], *n_m_* = 3.4 (silicon), *n_s_* = 1.35 (PBS), *S* = 500 nm/RIU [41], *δλ* = 0.4 nm and *λ* = 1,550 nm, the bound surface density is found to be 5 × 10^8^ molecules/mm^2^. For avidin, this corresponds to a surface mass density of 60 pg/mm^2^. From FDTD simulations of the cavity, the sensing surface area is estimated to be as low as 2.2 μm^2^ (based on the surface area of the slot walls within, and the six holes surrounding, the cavity), thus the bound mass detected is of the order of 100 ag. Such numbers should be used with caution, as they are based on assumptions. The concentration limit of detection is the most reliable quantity as this was actively controlled; it should be noted, however, that this value will differ for different antibody-antigen pairs. Although a biotin solution is left in contact with the substrate for several hours in order to ensure saturation, and the specificity of this protocol has already been established [1], non-specific binding could be eliminated by adding suitable blocking agents to the protocol.

This result highlights the increased light-matter interaction of slotted photonic crystal cavities. Wavelength shifts comparable to that of much larger structures can be observed in smaller devices. Compactness allows potential for hundreds of these devices to be integrated onto a centimetre scale chip. When combined antibody patterning techniques, this would allow hundreds of independent biological tests to be conducted simultaneously using a minimum amount of precious analyte. The disadvantage when compared with larger structures is that the target sensing area is much smaller. When dealing with very low concentrations, the target molecule may never see the 2.2 μm^2^ sensing area on a reasonable timescale. This is a general limitation of all surface affinity based biosensors. Microfluidics has potential to help here. Water absorption at 1,550 nm also impacts on the performance of the device, by reducing the quality factor of the cavity. High quality factor cavities are advantageous for avidin sensing as higher Q-factor cavities allow smaller shifts to be detected. The slot engenders higher field overlap with the sample, which gives bigger shifts but also results in greater absorption from the solution in which the target molecules are contained when compared to standard ring resonators. Shifting to shorter wavelengths, where water absorption is lower, should reduce this problem. 1,550 nm was chosen here due to the wide availability of sources and detectors and the group's familiarity with working at this wavelength. The lowest water absorption occurs at visible wavelengths, but this is inaccessible with slotted photonic crystals fabricated in silicon, due to strong absorption of silicon below 1,000 nm. Other materials (such as polymers) that are transparent at such wavelengths do not have as high a refractive index as silicon, and are thus not of sufficient index contrast necessary for the slot waveguide effect. Reducing the wavelength also requires downscaling the photonic crystal parameters (radius, slot width, period), which is harder to fabricate and increases potential for clogging. Despite these problems it is conceivable that performance could be improved by using wavelengths around 1,300 nm. The quality factor *Q* of a photonic crystal cavity is given by [102]:

(4) $$\frac{1}{Q_{\text{tot}}}=\frac{1}{Q_{\text{rad}}}+\frac{1}{Q_{\text{abs}}}$$

where the subscripts refer to the total Q-factor, and the Q-factors describing radiation and absorption losses. At the 1/e point, the propagation limit *L* due to absorption is given by:

(5) $$L=\frac{1}{\alpha }$$

where *α* is the absorption coefficient. The relationship between the quality factor and the lifetime *τ_c_* of a cavity is given by:

(6) $$Q_{\text{abs}}=\frac{2\pi f\tau_{c}}{\eta }$$

where *η* is the overlap of the mode with the absorbing medium and *f* is frequency. Combining Equations (5) and (6) yields the relationship between *Q_abs_* and *α*:

(7) $$Q_{\text{abs}}=\frac{2\pi n}{\alpha \lambda \eta }$$

where *n* is the refractive index of the cavity medium. As *α* ≈ 9.68 cm^−1^ and *n ≈* 1.319 at 1,550 nm [103], and the overlap of the mode with water within the slot is approximately 0.85, then Equation (7) gives a value of *Q_abs_* = 6,500. Taking *Q_rad_* ≈ 50,000 (highest Q in experiment without water [41]), then from Equation (4) *Q_tot_* = 5,700, which is somewhat higher but of the same order, as the maximum experimental Q-factor we observe (*Q_exp_* ≈ 4,000 [41]). The water absorption at 1,300 nm is almost one order of magnitude lower as is evident from its absorption coefficient, *α* ≈ 1.34 cm^−1^ [103]. Using the same analysis as before, for 1,300 nm, given that *n ≈* 1.324, the quality factors are predicted to be *Q_abs_* = 56, 000 and *Q_tot_* = 26,000. As this Q-factor is one order of magnitude greater than at 1,550 nm, working at 1,300 nm should allow smaller shifts to be detected.

## 7. Barriers to Entry

From the wide variety of applications, sensitivity and commercial success, surface plasmon resonance can be viewed as the technology to beat in terms of label-free biosensing. For any technology to replace the existing standard it must meet at least one of the following requirements:

**Perform better:** in the case of biosensing, having better sensitivity or limit of detection, and/or be easier to operate.
**Cost less:** For biosensors, not only the cost of the device, but also the cost per test in terms of materials and labour.
**Do something new:** can the biosensor investigate some problem that other devices cannot?

One of the main barriers for taking full advantage of the integrated optics (ring resonator, photonic crystal, slot waveguide *etc*.) approach is the sources and detectors available. Although the sensor chips can be very small, they often require a bulky spectrometer to function fully, and are thus more ‘chip-in-a-lab’ than ‘lab-on-a-chip’. This point is often brushed over and is one of the key limiting factors of many devices in the literature. Multiplexing at the same time can also be tricky. Either the source has to be split and the detector arrayed (e.g., [18]), which can be lossy, or each sensor has to be rapidly addressed individually as in the case of Genalyte [36]. Sensors based on silicon also suffer from a lack of integrated light sources due to the indirect bandgap of silicon, though solutions such as flip-chip bonding can be used to attach sources constructed in other material platforms. A further barrier comes from the fact that the machines sold by Biacore benefit from a research and commercial history stretching back more than 20 years, and possess the majority of the current market.

More generally the fragile and expensive nature of antibodies limits the vision of protein microarrays below their DNA counterparts. Whilst DNA can be copied rapidly in PCR, proteins are more vulnerable to harsh conditions. The use of surface based sensors also means that detection is based on diffusion and sedimentation, which limits both performance and speed. Antigens in solution may never see the capture antibody on the surface of the device within a reasonable timescale. Microfluidics has potential to help here. Many photonic sensors also rely on having prepared solutions involving steps such as centrifugation and dilution rather than work with unaltered samples (e.g., a drop of blood), and are thus not user-friendly to the non-specialist. Despite all of these problems, it is conceivable that integrated optical sensors will feature in future commercial biosensors due to the advantages of size, mass producibility and sensitivity they offer.

## 8. Summary and Outlook

Slotted photonic crystals have been developed towards a realistic and promising platform for lab-on-a-chip style biosensors. The great advantage over many other photonic devices is the strong confinement of light, not only spatially, but also temporally due to combination of slot waveguide and photonic crystal effects. To better understand the limitations of the system and to maximise the amount of light within the slot, we performed studies on the losses. The propagation losses were found to be comparable to standard photonic crystals in the fast regime as a result of the slot waveguide effect. Coupling losses were reduced to 1.5 dB per interface over a large bandwidth of 78 nm using a resonant coupler design. This allowed full advantage of the slotted photonic crystal light confinement in air to be taken. The sensitivity of this system was shown in proof-of-principle experiments where the protein avidin was detected, at a lowest concentration of 15 nM, and where bigger shifts in resonant wavelength were observed for the same concentration as many larger photonic biosensors. When combined with the very small size of the device, this makes them attractive for the high throughputs, multiplexing and multifunctionality required by protein microarrays. Larger wavelength shifts also reduce the performance requirements for any on-chip spectrometer, which is necessary for a real lab-on-a-chip device. This work also showed that slotted photonic crystals can be integrated with microfluidic circuits.

Slotted photonic crystals therefore provide an entirely new platform for optical biosensing, allowing higher sensitivities in smaller structures, and great potential for on-chip spectrometry. Whilst it will take at timeframe of 5–10 years and more resources and engineering to make this a commercial reality, the basis of design, performance, detection, proof-of-principle and application have all been demonstrated within this body of work. Slotted photonic crystals therefore still have much more to give, and have strong potential for true ‘lab-on-a-chip’ devices. Our hope is that with this foundation they will shine new light on many of the mysteries of biology in years to come.

## Figures and Tables

**Figure 12.**
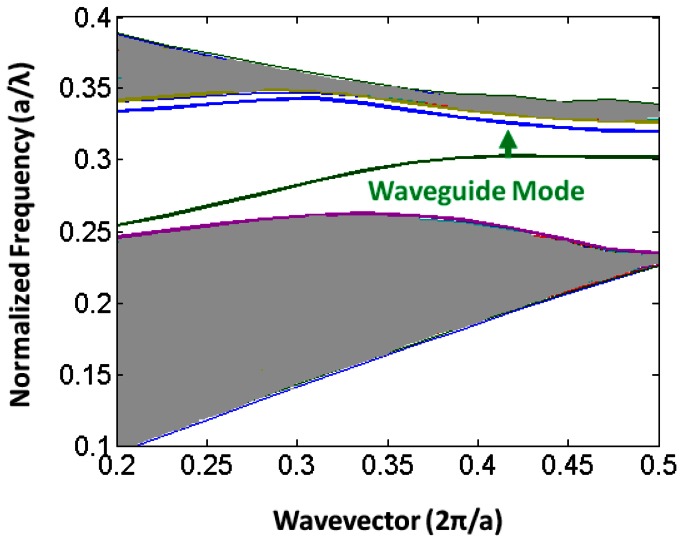
Bandstructure of slotted photonic crystal waveguide showing waveguide mode. A small local compression of the lattice period reduces the amount of dielectric, which in turn raises the operational frequency further into the forbidden bandgap region.

**Table 1.** Comparison of selected small optical biosensors.

**Reference**	**Type of Device**	**Target Substance**	**Estimated Sensing Surface Area (**μ**m^2^)**	**Minimum Conc. Measured**	**Δλfor 10** μ**g/mL Conc. (nm)**
**Slot Waveguide**					
Barrios *et al.* [16]/ Banuls *et al.* [17]	Slot ring resonator	BSA	>250	0.042 μg/mL	2.5
Carlborg *et al.* [18]	Slot ring resonator	Anti-BSA	>250	0.125 μg/mL	1.2
Claes *et al.* [19]	Slot ring resonator	Avidin	>13	100 μg/mL (1.5 μM)	-
**Photonic Crystal**					
Zlatanovic *et al.* [24]	2-D Photonic crystal cavity	Anti-biotin	0.272	20 pM	-
Toccafondo *et al.* [20]	2-D Photonic crystal waveguide	s-DNA	>100	19.8 nM	< 0.2
Skivesen *et al.* [21]	2-D Photonic crystal waveguide	BSA	>100	10 μg/mL (150 nM)	0.2
Dorfner *et al.* [25]	2-D Photonic crystal cavity	BSA	8.15	<1 μM	-
Lee and Fauchet [22,23]	2-D Photonic crystal cavity	BSA	50	2%	-
Kang *et al.* [26]	2-D Photonic crystal cavity	3-APTES	-	-	-

Guo *et al.* [29]	1-D Photonic crystal	Biotin-20T	-	1 nM	0.5
Orosco *et al.* [30]	1-D Porous silicon photonic crystal	Protease	-	7 μM	-
Mandal *et al.* [27]	1-D Photonic crystal cavity	Anti-streptavidin	8.36	1 μg/mL	0.25
Konopsky and Alieva [28]	1-D Photonic crystal	Biotin	-	3 μg/mL	-
**Slotted Photonic Crystal**					
Scullion *et al.* [42]	Slotted photonic crystal cavity	Avidin	<2.2	1 μg/mL (15 nM)	1.5
Lee *et al.* [51]	Slotted microcavity photonic crystal	Latex spheres	<2	-	-

Kita *et al.* [50]	Slotted photonic crystal nanolaser	BSA	<1	255 fM	-

Lai *et al.* [45]	Slow light slotted photonic crystal	Xylene in water	-	86 μg/mL	N/A

**Table 2.** Propagation loss comparison.

**Reference**	**Device**	**Material**	**Slot Width (nm)**	**Propagation Loss (dB/cm)**
Di Falco *et al*. [78]	Slotted photonic crystal	Silicon	115	29 ± 10
			125	9 ± 5
			135	4 ± 7
			145	8 ± 6
Baer-Jones *et al*. [80]	Slot waveguide ring resonator	Silicon	50	11.6 ± 3.5
			60	7.7 ± 2.3
			70	8.1 ± 1.1
Spott *et al*. [81]	Asymmetric slot waveguides	Silicon/PMMA	130	1.7 ± 1.1
Zhang *et al*. [82]	Slot waveguide	Silicon	80	8.6 ± 0.61
			100	11.1 ± 1.15
Alasaarela *et al*. [83]	Slot waveguide	Silicon/TiO2	Uncoated	71
			Coated	7 ± 2
Notomi *et al*. [79]	Standard photonic crystal waveguide	Silicon	N/A	2
